# Social capital and chemsex initiation in young gay, bisexual, and other men who have sex with men: the pink carpet Y cohort study

**DOI:** 10.1186/s13011-021-00353-2

**Published:** 2021-02-19

**Authors:** Rayner Kay Jin Tan, Caitlin Alsandria O’Hara, Wee Ling Koh, Daniel Le, Avin Tan, Adrian Tyler, Calvin Tan, Chronos Kwok, Sumita Banerjee, Mee Lian Wong

**Affiliations:** 1 Saw Swee Hock School of Public Health, National University of Singapore, 12 Science Drive 2, MD1 Tahir Foundation Building #10-01, Singapore, 117549 Singapore; 2 Yong Loo Lin School of Medicine, National University of Singapore, 10 Medical Dr, Singapore, 117597 Singapore; 3 Action for AIDS Singapore, 9 Kelantan Lane #03-01, Singapore, 208628 Singapore; 4 National University Hospital, National University Health System, Singapore, Singapore

**Keywords:** Chemsex, Gay men, MSM, Singapore, Social capital

## Abstract

**Background:**

Young gay, bisexual, and other men who have sex with men (YMSM) are especially vulnerable to the risks associated with sexualized substance use, or ‘chemsex’. Engaging in chemsex established as a major risk factor for Human Immunodeficiency Virus (HIV) acquisition, and is thus a public health issue of increasing urgency. This paper attempts to explore the association between measures of social capital and patterns of sexualized substance use among a sample of YMSM in Singapore.

**Methods:**

Results of this study were derived from baseline data of the Pink Carpet Y Cohort Study in Singapore, comprising a sample of 570 HIV-negative YMSM aged 18 to 25 years old. Latent class analysis was employed to identify classes with similar patterns of sexualized substance use, and multinomial logistic regression was employed to examine associations between class membership and proxy measures of social capital, including age of sexual debut, bonding and bridging social capital, connectedness to the lesbian, gay, bisexual and transgender community, and outness.

**Results:**

Latent class analysis revealed three classes of YMSM based on their histories of sexualized substance use, which we labelled as ‘alcohol’, ‘poppers’, and ‘chemsex’. Multivariable analyses revealed that participants who were older (aOR = 1.19, *p* = 0.002) and who identified as gay (aOR = 2.43, p = 0.002) were more likely to be in the poppers class compared to the alcohol class. Participants with a later age of sexual debut were increasingly less likely to be in the poppers (aOR = 0.93, *p* = 0.039) and chemsex classes (aOR = 0.85, *p* = 0.018), compared to the alcohol class.

**Conclusions:**

Varying measures of social capital such as an earlier age of exposure to sexual networks may predispose YMSM to greater opportunities for sexualized substance use. Future interventions should target YMSM who become sexually active at an earlier age to reduce the risks associated with sexualized substance use.

## Introduction

Gay, bisexual, and other men who have sex with men (GBMSM) constitute a key population that continues to be disproportionately affected by Human Immunodeficiency Virus (HIV) on a global scale [1]. Within communities of GBMSM, young gay, bisexual, and other men who have sex with men (YMSM) experience a greater burden of HIV risk compared to their older counterparts, which may be attributed to a higher incidence of risky sexual behaviors, sexualized substance use, and barriers to health-seeking behaviors [2, 3].

The use of amphetamine-type stimulants (ATS), in particular methamphetamine, has become increasingly prevalent among GBMSM [4]. Methamphetamine is a derivative of amphetamine that is often used by GBMSM in sexualized contexts, known colloquially as ‘chemsex’ [5]. Other drugs typically associated with chemsex include gamma-hydroxybutyrate/gamma-butyrolactone (GHB/GBL), mephedrone, ecstasy, ketamine, as well as drugs typically prescribed for erectile dysfunction (ED) such as Viagra or Cialis [6, 7]. Chemsex has been identified in numerous studies as a significant risk factor for HIV among GBMSM due to the interplay between drug use and behaviors that place such individuals at a higher risk of acquiring HIV, including unprotected anal sexual intercourse with an increased number of sex partners, prolonged sexual encounters, and the sharing of sex toys [8, 9].

Globally, the trend of amphetamine-type stimulant use remains high among GBMSM. A 2012 study among GBMSM in 12 countries across Asia reported ATS to be the most widely used types of drugs among the 10,861 participants sampled, with 8.1% reporting to have recently used ecstasy and 4% having used methamphetamine [10]. This trend of ATS use is also reflected in non-Asian settings [4, 11, 12]. YMSM are also especially vulnerable to substance use disorders and the risks associated with them. A 2014 study conducted among 595 YMSM aged 12 to 24 years old in across eight cities in the United States found that 10.8% of participants surveyed has reported using methamphetamine in the past 90 days [13]; other studies have similarly reported greater instances of substance abuse among YMSM relative to older cohorts of GBMSM [14–16].

The use of amyl nitrites, otherwise known as ‘poppers’, in sexual contexts is has been reported to be common as well for GBMSM across various settings largely as a means of sexual enhancement [17–19]. Popper use has been found to be associated with HIV seroconversion as well as sexual risk behaviors such as having multiple sex partners and having unprotected anal intercourse [20–22]. Demographic and psychosocial correlates for popper use among GBMSM include being older, being HIV-positive, and reporting visiting sex-on-premises venues, licensed lesbian, gay, bisexual, and transgender (LGBT) venues, and using other substances [19, 23].

Apart from the use of typically illicit substances, heavy alcohol use has also been established to be common among GBMSM, especially in developed country settings [24–26]. Past studies have also found that individuals who identify with any sexual minority status, including GBMSM, had initiated alcohol use at a younger age, compared to their heterosexual counterparts [27, 28]. An early onset of alcohol initiation among GBMSM has been found to be associated with a greater number of lifetime sexual partners, elevated levels of depressive symptoms, and alcohol abuse later in life [29]. Heavy alcohol use among GBMSM, and in particular the use of alcohol during or after sex, has also been found to be associated with behaviors associated with HIV acquisition risk, such as unprotected anal intercourse [30–32]; however, these findings have been inconsistent due to methodological differences in measuring alcohol use and the lack of experimental study designs [33].

### Risk factors for substance use

Risk factors for substance use in general, including the use of ATS among GBMSM, have been well-established in the extant literature. Depression severity is a risk factor that increases individuals’ likelihood of using ATS, with participants reporting that the use of substances help to alleviate the negative effects of such mood disorders [34]. Additionally, MSM who experience consistent episodes of neglect or trauma at a young age also report increased susceptibility of eventual methamphetamine use [35]. Other studies have qualitatively evaluated that methamphetamine use is perceived to be instrumental in alleviating personal dread of growing old and sickly, or fears of becoming less attractive and unwanted [36]. A recent study in Singapore found that GBMSM engaged in chemsex as a means of coping with societal rejection [7]. Methamphetamine use, especially in the context of chemsex, has also been established as an important means of providing a safe haven from sexual discrimination and isolation among GBMSM living with HIV [37].

Reasons for heavy alcohol use reported by GBMSM in general may include life histories of traumatic experiences such as sexual orientation-based discrimination and childhood sexual abuse [38, 39], while YMSM specifically reported social drinking motivations as a main reason for engaging in heavy alcohol use [40]. As for the use of alcohol during sex among GBMSM, reasons included more situational or contextual factors such as the facilitation of cognitive ‘escape’ for the awareness of HIV risk, or the enhancement of sexual pleasure [41, 42].

### Situating social capital as risk factors for sexualized substance use

Adequate social support has been credited in various studies as one of the main reasons for the reduction in risky sexual behaviors among GBMSM [43]. Specifically, community connectedness plays an instrumental function in the provision of intangible support, especially for GBMSM who may experience difficulties in tapping on their kinship or peer network for social support, or who have repressed their sexuality out of fear of being discriminated [44, 45]. However, other studies have reported a positive association between drug use and GBMSM community engagement as well (Wei et al., 2012); findings from studies pertaining to the relationship between of community connectedness and personal forms of social capital on drug use among GBMSM thus remain mixed. These mixed findings may be attributed to the varying ways in which community affiliation or social capital have been measured or conceptualized in various studies, which may span across measures that reflect access to social networks, psychological feelings of affiliation, or even explicit forms of affiliation that presuppose participation in interest groups or activities.

With regard to alcohol use, GBMSM who report a stronger affiliation with gay male culture and who have met sexual partners through entertainment establishments where alcohol use takes place are more likely to have engaged in heavy alcohol use [24, 26]. Among YMSM, gay bar attendance, depression, sensation seeking, peer risk behaviors, multiple sexual partners, and a younger age of alcohol initiation were found to be associated with heavier alcohol use patterns [46, 47].

There has been a lack of consensus around the conceptualization, operationalization, and thus measurement of social capital. This has resulted in a large variety of ways, sometimes conflicting, in which measures that constitute social capital have been linked to substance use-related behaviors among GBMSM. Past studies that measure varying forms of social capital and its relationship with substance use among GBMSM have tended to measure only one form or dimension of social capital, or conflate different nuanced aspects of social capital with one another, which have led to conflicting findings in some cases.

Measures of the *social cohesion* school of social capital, conceptualized as the resources available to members or citizens of a social group or society and as a property that exists at the group level, are relevant to individual health outcomes as an individual may benefit from being embedded or immersed in a given context. These group level attributes may include group-aggregated, contextual measures such as perceptions of trustworthiness and collective socialization (i.e. adults in community and not just parents shape child development) [48]. On the other hand, measures of the *network theory* approach, conceptualized as the resources that are embedded within an individual’s social networks, include both egocentric (i.e. individual-centred) and sociometric (i.e. group-centred) properties. At the egocentric level, these may include measures that reflect the functional nature of ties with others in the community, such as measures of social support and those that reflect the availability of necessary resources, or measures that reflect the structural nature of ties, such as the size of one’s own network or the density of relationships and ties within those networks [49].

In the context of risk factors for substance use among GBMSM, measures of community connectedness, or a psychological feeling of being connected to the wider community, may align more with the social cohesion approach to social capital. On the other hand, measures that reflect an individual’s access to social support from others may reflect the functional nature of ties within a network, whereas one’s access to substances via other substance users may reflect more structural aspects of one’s own network. In this study, we selected several potential measures of social capital that we thought were potentially epidemiologically relevant as risk factors for substance use among YMSM in Singapore, including age of sexual debut (as a proxy for one’s initiation into sexual networks, and thus access to sexualized substance use), bonding social capital, bridging social capital, connectedness to the LGBT community, as well as the extent of sexual orientation disclosure or ‘outness’ to family, to the world, and to religion, as a proxy for being able to access social support, given major barriers such as prevailing stigma and the criminalization of sexual relations between men in the present setting.

### Substance use among GBMSM in Singapore

As of 2019, a total of 8295 incident HIV infections have been notified with the ministry of health in Singapore (MOH). The spread of HIV in Singapore is characterized by its concentration among GBMSM, as well as older, heterosexual men [50]. Despite being an established risk factor for HIV and other STI acquisition, few studies have attempted to study patterns of drug use among GBMSM in Singapore, notwithstanding the Asia Internet MSM Sex Survey (AIMSS) that was conducted from 2009 to 2010 among 4072 GBMSM from Singapore, which found that 12.8% of participants had reported consuming drugs prior to, or during sex in the preceding six months [51]. A more recent qualitative study among GBMSM in Singapore found that chemsex was perceived to be common in Singapore, and openly solicited through geosocial networking apps [7].

Social capital among GBMSM in Singapore should be interpreted in light of the sociocultural milieu in which they are embedded. Singapore society has largely held negative perceptions of, and attitudes towards lesbian, gay, bisexual, and transgender (LGBT) individuals [52–54], as well as people who use drugs [55]. Criminal legislation towards sexual minorities and people who use drugs have corresponded, and arguably, contributed, to these negative attitudes. Section 377A of the Singapore Penal Code criminalizes consensual sexual behaviour between men, with penalties for imprisonment for a term that may extend up to no longer than two years. Drug use and trafficking of drugs are also punished by severe penalties under the Singapore Penal Code. The Misuse of Drugs Act criminalizes the possession and use of drugs with penalties that range from fines of up to S$20,000 to a maximum of ten years in prison, and trafficking of drugs beyond stipulated thresholds with a mandatory death penalty or life imprisonment.

### Rationale and aims of study

The aims of this study are two-fold. Firstly, given the risks associated with substance use, coupled with preliminary data on the rise in the incidence and prevalence of chemsex among GBMSM in Singapore, we embarked on this study to address the dearth of information on the risk factors associated with chemsex among YMSM in Singapore. Secondly, mixed findings around the relationship between measures of social capital and substance use in general may be attributed to the varying ways in which community affiliation or social capital have been measured or conceptualized in various studies, which may span across measures that reflect access to social networks, psychological feelings of affiliation, or even explicit forms of affiliation that presuppose participation in interest groups or activities.

To fill this gap, this study sought to firstly identify candidate variables through our initial literature review, as well as in consultation with our community partner through which this study was conducted, Action for AIDS Singapore (AFA). These measures were chosen to reflect potential risk factors identified by members of the local GBMSM community, as well as to advance our knowledge of the link between some of these measures that have been employed in other health-related studies, but not in the context of chemsex. We balanced the need for conciseness in our eventual survey, and decided on measures of social capital that included age of sexual debut, bonding social capital, bridging social capital, community connectedness, as well as the extent of sexual orientation disclosure or ‘outness’ to varying social groups, on its association with varying patterns of substance use. This approached would allow us to draw more nuanced, exploratory claims around the role of social capital and its potential relationship with varying patterns of substance use among YMSM.

## Methods

### Participants and recruitment

The Pink Carpet Y Cohort Study is Singapore’s first prospective cohort study exploring the syndemic risks associated with HIV and other sexually transmitted infections (STI) acquisition among YMSM. This study was a partnership between AFA, one of Singapore’s longest-running community-based organizations serving the health of GBMSM, and the Saw Swee Hock School of Public Health, National University of Singapore. To be eligible for this cohort, participants had to be HIV-negative or unsure of their HIV status, between the ages of 18 to 25 years old, Singapore citizens or permanent residents, and identify as gay, bisexual, or queer men at the point of recruitment, which spanned across May to September 2019. Participants were asked to self-report these attributes.

Participants were invited to participate in this study through a recruitment flyer that was disseminated through both online (e.g. social media) and offline (e.g. at the organization’s office or outreach areas) channels by a network of community-based organizations in Singapore who are engaged in health advocacy-related activities for GBMSM. Participants who were interested in participating and were eligible for the study signed up through an enrolment link with their self-reported alias, contact details, date of birth, gender, HIV status, sexual orientation, and their residence status. An AFA staff member subsequently verified the eligibility of participants who had signed up prior to sending them a unique identifier, and a link for the baseline survey.

Participants were provided with a Singapore Dollars (SGD) 20.00 (approximately United States Dollars [USD] 15.00) cash reimbursement upon completion of the baseline survey. A total of 570 participants were recruited at the baseline of the cohort, however the response rate could not be established as it was not possible to ascertain the total number of eligible participants that the recruitment flyers had reached. Participants could also refer their friends to participate in the survey and be reimbursed SGD5.00 (approximately USD3.75) for each friend successfully referred and who had completed the baseline survey; a total of 171 (30.0%) of participants were recruited through referrals.

### Ethics declaration

Ethics approval was obtained from the institutional review board at the National University of Singapore (Reference Code S-19-007) prior to data collection.

### Variable measures

The survey collected sociodemographic information from respondents, including age (in years), ethnicity (Chinese vs non-Chinese), gender (cisgender vs non-cisgender), sexual orientation (gay vs non-gay), and monthly household income (SGD5000 and above vs below SGD5000; SGD5000 is approximately USD3668.94). As the YMSM in our sample included respondents who were still in school, educational attainment and gross monthly personal income were omitted as variables, though they were collected in the baseline survey. Household income was chosen as a proxy variable for socioeconomic status among participants, given that most participants were not expected to report a personal monthly income that would reflect the socioeconomic strata in which they were embedded. We collected self-reported histories of the use of alcohol, poppers, methamphetamine, GHB/GBL, and ED drugs in sexual contexts, which were coded as a categorical variable (yes vs no).

Age of sexual debut was measured through a series of questions that asked respondents about their age when they first had either insertive or receptive oral or anal sex with another man. Personal social capital was measured through the brief 16-item personal social capital scale (PSCS-16) validated by Wang and colleagues [56, 57]. Participants were asked to describe, through a five-point Likert scale, the perceived quantity and quality of their relationships with individuals and organizations; 1 being *none* or *a few* and 5 being *all* or *a lot.* The scale was initially developed to measure two distinct forms of social capital; *bonding* and *bridging* social capital. Putnam [58] conceptualized individual social capital across two distinct forms; bonding and bridging social capital, which are defined as the existence of within-group and between-group network ties, respectively. The first eight items on the PSCS-16 were designed to measure bonding social capital, while the last eight items measured bridging social capital. Both bonding and bridging social capital were measured as indices that were thus the sum score of eight items, with a minimum score of 8 and a maximum score of 40. Cronbach’s alpha of 0.77 and 0.89 were reported for the bonding social capital and bridging social capital subscales, respectively.

Connectedness to the LGBT community was an eight-item scale adapted from Frost and Meyer [59]. Items in the scale were amended slightly to suit the present setting; for example, the statement “you feel you’re a part of NYC’s LGBT community” was amended to “You feel you’re a part of Singapore’s LGBT community”. Each item was measured through a four-point Likert scale from 1 to 4, with 1 being that they *strongly disagree* and 4 being that they *strongly agree*. Community connectedness was measured as an index that was the sum score of all eight items, with a minimum score of 8 and a maximum score of 32. Cronbach’s alpha of the scale was reported as 0.86.

Outness was measured through the outness inventory, a 10-item scale developed by Mohr and Fassinger [60]. The outness inventory assesses the degree or magnitude to which lesbian, gay, and bisexual individuals are open or ‘out’ about their sexual orientation to other individuals. Questions asked in this scale required participants to think about how different individuals in their own social networks (e.g. mother, siblings, religious leaders etc.) may know about, or openly talk about the participant’s own sexual orientation. Participants could select responses from 1 to 7, with 1 being that *the person definitely does not know about your sexual orientation* and 7 being that *the person definitely knows about your sexual orientation, and it is openly talked about*. The overall outness score was calculated as an average of three subscales, including *outness to family*, *outness to religion*, and *outness to the world*. Cronbach’s alpha of the scale was reported as 0.82. The subscales were used in analysis instead of the overall scale as a means of investigating how outness to varying groups may affect the chosen outcome variable.

### Statistical analyses

Statistical analysis was carried out using the statistical software STATA version 15 (Stata Corp, College Station, TX, USA). Latent class analysis (LCA) was performed with STATA’s *gsem* function to delineate classes of drug use in sexualized contexts among participants who ever had a sexual experience (*n* = 511), which formed our analytic sample for further analyses. The chosen variables included a history of using alcohol, poppers, meth, GHB/GBL as well as other ED medication or drugs (e.g. Viagra, Cialis, ‘black ants’) in sexualized contexts. A latent class model was employed, whereby conditional item probabilities for each class, as well as class probabilities were estimated through maximum likelihood procedures. A posteriori probabilities were calculated using the *predict* post-estimation command. Models were compared using Bayesian information criteria (BIC) given its reliance on both the log-likelihood and the adjusted sample size [61], and a three-class model had the lowest BIC. We opted to present our findings through a latent class model so that the findings would be more easily translatable through differentiated models of care, and interpreted by policymakers in the context of the evidence that already exists on chemsex among GBMSM in Singapore.

We employed descriptive and bivariate statistics to identify trends, and differences between such trends across subgroups, respectively. Multinomial logistic regression was employed to examine the association between respondents’ sociodemographic attributes and social capital measures with their sexualized substance use class membership. Multicollinearity was assessed through investigating variance inflation factors. Statistical significance was set at *p* < 0.05.

## Results

### Sociodemographic attributes and description of overall sample

A total of 570 participants were recruited in this study. Table 1 summarizes the sociodemographic attributes and overall description of the overall sample. In terms of their sociodemographic attributes, the mean age of the sample was 21.9 years (Standard Deviation [SD] = 2.17). 83.9% of the participants identified as Chinese (*n* = 478), 92.1% identified as cisgender male (*n* = 525), 71.6% identified as gay (*n* = 408), and 35.6% reported a monthly household income of SGD5000 and above (*n* = 203). A total of 66.7% (*n* = 190), 28.3% (*n* = 161), 4.7% (*n* = 27), 4.7% (n = 27) and 4.6% (*n* = 26) reported ever using alcohol, poppers, methamphetamine, GHB/GBL, and ED medication in sexual contexts, respectively. Participants reported an average age of sexual debut at 17.3 years old (SD = 2.94), and 511 out of 570 participants (89.6%) ever had sex in the sample. Participants scored an average of 21.7 (SD = 4.97) for the bonding social capital subscale and 18.2 (SD = 5.45) for the bridging social capital subscale. An average score of 22.4 (SD = 4.33) was reported for the connectedness to the LGBT community scale. Finally, participants reported an average of 2.7 (SD = 1.60), 0.7 (SD = 1.27), and 2.6 (SD = 1.74) on the outness to family, religion, and the world subscales, respectively.

**Table 1:** Sociodemographic attributes and description of sample (*n* = 570)

Demographic Variables	n	%	Mean	SD
Age			21.9	2.17
**Ethnicity**				
Chinese	478	83.9%		
Non-Chinese	92	16.1%		
**Gender**				
Cisgender male	525	92.1%		
Transgender, genderqueer, or others	45	7.9%		
**Sexual orientation**				
Gay	408	71.6%		
Bisexual, queer, or others	162	28.4%		
**Monthly household income**				
SGD 5000 and above	203	35.6%		
Below SGD 5000	367	64.4%		
**Ever had sexualized alcohol use**				
Yes	190	66.7%		
No	380	33.3%		
**Ever had sexualized popper use**				
Yes	161	28.3%		
No	409	71.8%		
**Ever had sexualized methamphetamine use**				
Yes	27	4.7%		
No	543	95.3%		
**Ever had sexualized GHB / GBL use**				
Yes	27	4.7%		
No	543	95.3%		
**Ever used erectile dysfunction drugs for sex**				
Yes	26	4.6%		
No	544	95.4%		
**Age of sexual debut (n = 511)**			17.3	2.94
**Bonding social capital**			21.7	4.97
**Bridging social capital**			18.2	5.45
**Connectedness to LGBT community**			22.4	4.33
**Outness to family**			2.7	1.60
**Outness to religion**			0.7	1.27
**Outness to the world**			2.6	1.74

Abbreviation: SD, Standard Deviation; GHB/GBL, gamma-hydroxybutyrate/gamma-butyrolactone; LGBT, Lesbian Gay Bisexual Transgender

### Social capital attributes by sexualized substance use class membership

LCA revealed three classes, which we labelled post hoc as ‘*alcohol’*, ‘*poppers’*, and ‘*chemsex’*. Table 2 describes the demographic and social capital attributes of participants by class membership. Participants in the alcohol class (*n* = 348) reported only ever using alcohol and never using any other substances during sex. Participants in the poppers class (*n* = 140) had mostly ever used poppers during sex (*n* = 136, 97.1%), with some reporting ever using other chemsex-related substances during sex. Most participants in the chemsex class (*n* = 23) reported using methamphetamine, GHB/GBL, and ED drugs during sex.

**Table 2:** Sociodemographic attributes and description of analytic sample by substance-using classes (n = 511)

Demographic Variables		Alcohol (n = 348)		Poppers (***n*** = 140)		Chemsex (***n*** = 23)		Test for difference*
		n/Mean	%/SD	n/Mean	%/SD	n/Mean	%/SD	
**Age**		21.8	2.15	22.5	2.10	22.3	1.77	0.005
**Chinese ethnicity (Ref = Non-Chinese)**		292	83.2%	121	86.7%	18	78.3%	0.499
**Cisgender male (Ref = Transgender, genderqueer, or others)**		320	92.0%	136	97.1%	21	91.3%	0.066
**Gay (Ref = Bisexual, queer, or others)**		239	68.7%	121	86.4%	18	78.3%	< 0.001
**Monthly household income ≥ SGD5000 (Ref < SGD5000)**		112	32.2%	63	45.0%	7	30.4%	0.025
**Ever had sexualized substance use with**								
Alcohol		89	25.6%	80	57.1%	13	56.5%	< 0.001
Amyl nitrites (Poppers)		0	0.0%	136	97.1%	22	95.7%	< 0.001
Crystal methamphetamine		0	0.0%	5	3.6%	22	95.7%	< 0.001
GHB/GBL		0	0.0%	5	3.5%	22	95.7%	< 0.001
Erectile dysfunction drugs		0	0.0%	9	6.3%	17	73.9%	< 0.001
**Age of sexual debut**		17.5	2.89	17.1	3.13	16.2	2.33	0.078
**Bonding social capital**		21.4	5.02	22.7	4.61	22.0	6.48	0.028
**Bridging social capital**		18.2	5.56	18.3	4.89	18.4	6.76	0.868
**Connectedness to LGBT community**		22.4	4.30	22.0	3.98	21.5	5.04	0.443
**Outness to family**		2.6	1.60	2.9	1.51	3.3	1.73	0.028
**Outness to religion**		0.6	1.04	0.8	1.58	0.5	1.27	0.154
**Outness to the world**		2.5	1.64	3.0	1.92	3.0	2.28	0.011

Abbreviation: SD, Standard Deviation; GHB/GBL, gamma-hydroxybutyrate/gamma-butyrolactone; LGBT, Lesbian Gay Bisexual Transgender

*Fisher’s Exact Test were employed for categorical variables while One-Way ANOVA was employed for continuous variables

### Factors associated with substance-using class membership

Table 3 summarizes the multinomial logistic regression models with adjusted odds ratios (95%CI) for sexualized substance use class membership. Respondents who reported a later age of sexual debut were increasingly less likely to be in the poppers (aOR = 0.93, *p* = 0.039) and chemsex classes (aOR = 0.85, *p* = 0.018), compared to the alcohol class. Respondents who were older (aOR = 1.19, *p* = 0.002) and who identified as being gay as opposed to being bisexual or queer (aOR = 2.43, p = 0.002) were more likely to be in the poppers class, compared to the alcohol class.

**Table 3:** Multinomial logistic regression with adjusted odds ratios (95%CI) for sexualized substance use class membership (n = 511)

	Alcohol	Poppers			Chemsex		
		aOR	95% CI	p-value	aOR	95% CI	p-value
**Age**	Ref	**1.19**	**(1.07–1.32)**	**0.002**	1.18	(0.95–1.47)	0.134
**Chinese ethnicity (Ref = Non-Chinese)**	Ref	1.12	(0.61–2.05)	0.726	0.64	(0.21–1.92)	0.427
**Cisgender male (Ref = Transgender, genderqueer, or others)**	Ref	2.46	(0.79–7.62)	0.119	0.93	(0.18–4.69)	0.927
**Gay (Ref = Bisexual, queer, or others)**	Ref	**2.43**	**(1.39–4.27)**	**0.002**	1.74	(0.58–5.25)	0.324
**Monthly household income ≥ SGD5000 (Ref < SGD5000)**	Ref	1.51	(0.98–2.33)	0.064	0.91	(0.34–2.41)	0.848
**Age of sexual debut**	Ref	**0.93**	**(0.86–1.00)**	**0.039**	**0.85**	**(0.74–0.97)**	**0.018**
**Bonding social capital**	Ref	1.03	(0.98–1.09)	0.221	0.99	(0.89–1.10)	0.838
**Bridging social capital**	Ref	1.00	(0.95–1.04)	0.873	1.02	(0.93–1.12)	0.666
**Connectedness to LGBT community**	Ref	1.00	(0.95–1.05)	0.924	0.92	(0.83–1.02)	0.125
**Outness to family**	Ref	1.02	(0.88–1.18)	0.806	1.28	(0.97–1.68)	0.078
**Outness to religion**	Ref	1.07	(0.91–1.25)	0.445	0.89	(0.60–1.32)	0.555
**Outness to the world**	Ref	1.11	(0.98–1.26)	0.098	1.09	(0.85–1.40)	0.502

Notes

Abbreviation: CI, Confidence Interval; aOR, Adjusted Odds Ratio; LGBT, Lesbian Gay Bisexual Transgender

Statistically significant results at p < 0.05 are bolded

## Discussion

We classified participants based on their history of sexualized substance use for alcohol, poppers, methamphetamine, GHB/GBL and ED drugs through LCA. We took this approach to classifying participants by substance use patterns, and subsequently examining the associations between social capital measures with such class membership. One key finding of this study is that those with a later age of sexual debut were increasingly less likely to be in the poppers and chemsex classes of YMSM. We found that the aOR reported continued to decrease further towards the chemsex class of participants, which further strengthens the validity of this claim. This may be indicative of how exposure to sexual networks is a risk factor for using illicit substances typically associated with chemsex in Singapore, which corroborates past findings that such drugs are typically largely used in sexual contexts among YMSM [7, 8, 62]. This finding is unsurprising as earlier, and probably sustained exposure to sexually active GBMSM places one at greater risk of encountering substances such as methamphetamine or GHB/GBL. However, we were unable to ascertain if this effect is due to the duration of being exposed to such sexual networks, or the nature of the sexual relations that take place at a younger age that may be defined by greater power differentials between sexual partners.

Those who also identified as being gay (vis-à-vis bisexual or queer) or who were older were also more likely to be in the poppers class, compared to the alcohol class. This may firstly reflect the role that age may play in increasing an individual’s exposure to the possibility of substance use in any context, as well as the role that one’s sexual orientation may play in providing an individual access to certain social or sexual networks. These findings tie in with a possible narrative that a stronger sense of a gay male identity may be associated with greater participation in a wider gay male sexual culture that typically excludes GBMSM who do not identify as cisgender gay men [63, 64].

A higher level of connectedness to the LGBT community, which serves more as a measure of one’s psychological sense of connection to the wider LGBT community, appeared to be associated with a lower likelihood of being in the chemsex class compared to the alcohol class, although this was not statistically significant. This may tie in with evidence that argues for the role in which community connectedness plays in sexual identity formation and self-acceptance [65, 66], as well as buffering the effects of minority stress on well-being among GBMSM. However, our survey did not capture information on the frequency, intensity and routes of use for the varying substances and therefore cannot conclude if the types of substances used alone are indicative of greater levels stress, and warrants further study [67–69].

Being out to one’s family appeared to be associated with a greater likelihood of being in the chemsex class compared to the alcohol class, although this was not statistically significant. We hypothesize that this may be linked to minority stress as well, which may drive YMSM towards coping through substance use. Specifically, though sexual orientation remains a concealable identity [70], being out to one’s family results in YMSM’s sexual orientation no longer being concealed. This can lead to more minority stress for such YMSM who belong to families who stigmatize or discriminate them due to their sexual orientation. This may be further exacerbated by the largely negative views towards non-normative sexual orientations in Singapore. Further studies on the intersections of outness and minority stress are warranted to validate this claim.

We are mindful of several limitations of our study. As only HIV-negative YMSM were recruited in this study, we may have missed out on HIV-positive YMSM who are expected to report higher levels of substance use, which would be consistent with the findings of the other studies [37, 71]. Furthermore, as drug use carries with it severe penalties in Singapore, participants may not have been entirely honest with their answers around drug use, which may have led to an underreporting of substance use in the present sample. Finally, as multiple significance tests were conducted using *p*-value< 0.05, this may have resulted in an increased Type I error overall.

## Conclusions

This study among YMSM in Singapore found that participants with a later age of sexual debut were less likely to be in the poppers or chemsex class of YMSM, vis-à-vis the alcohol class. Our findings also indicate that while growing older in the gay community may lead to exposure to the possibility of substance use, an earlier age of sexual debut, and thus exposure to sexual networks at a younger age is a greater risk factor for chemsex among YMSM.

Moving forward, interventions could focus on providing YMSM a space to interact and navigate coming to terms with their sexual identities in safe and non-sexual settings. Due to prevailing stigma and the criminalization of sex between men, sexual health promotion and education for YMSM, as well as school-based programmes to address sexual health are not openly available in the present setting. The law criminalizing sex between men thus serves as a major barrier to providing support to YMSM, and should be reconsidered in light of the negative health implications that it may have for sexual minorities. In the absence of such reform, we recommend that NGOs be given the opportunity and resources to provide health promotion and education material and interventions for YMSM instead.

Efforts should also be taken to tackle stigma and a lack of understanding from Singaporeans at large, in order to facilitate YMSM more openly exploring their sexual identities, and lowering the barriers to accessing community-based resources or social support within the GBMSM or LGBT community. Specifically, given that being out to one’s family might subject YMSM to greater minority stress, efforts should be made to provide parents and families with support on how to healthily negotiate coming out experiences among children. With a more accepting social environment in Singapore, such offline communities would be far easier to form and sustain.

## Data Availability

The datasets used and/or analysed during the current study are available from the corresponding author on reasonable request.

## Abbreviations

**YMSM**: Young men who have sex with men
**HIV**: Human immunodeficiency virus
**LGBT**: Lesbian, gay, bisexual and transgender
**GBMSM**: Gay, bisexual and other men who have sex with men
**ATS**: Amphetamine-type stimulants
**GHB/GBL**: Gamma-hydroxybutyrate/gamma-butyrolactone
**ED**: Erectile dysfunction
**MOH**: Ministry of health
**STI**: Sexually transmitted infections
**AFA**: Action for AIDS Singapore
**SGD**: Singapore dollars
**USD**: United States dollars
**PSCS-16**: Personal social capital scale-16
**SD**: Standard deviation
